# Novel object recognition test as an alternative approach to assessing the pharmacological profile of sigma-1 receptor ligands

**DOI:** 10.1007/s43440-023-00516-x

**Published:** 2023-08-12

**Authors:** Katarzyna Szczepańska, Andrzej J. Bojarski, Piotr Popik, Natalia Malikowska-Racia

**Affiliations:** 1 Department of Medicinal Chemistry, Polish Academy of Sciences, Maj Institute of Pharmacology, Smętna 12, 31-343 Kraków, Poland; 2 Department of Technology and Biotechnology of Drugs, Faculty of Pharmacy, Jagiellonian University Medical College, Medyczna 9, 30-688 Kraków, Poland; 3 Department of Behavioral Neuroscience and Drug Development, Polish Academy of Sciences, Maj Institute of Pharmacology, Smętna 12, 31-343 Kraków, Poland

**Keywords:** Sigma-1 receptors, Functional profile, Novel object recognition test, Cognition, Memory impairment

## Abstract

**Background:**

Although the terms “agonist” and “antagonist” have been used to classify sigma-1 receptor (σ_1_R) ligands, an unambiguous definition of the functional activity is often hard. In order to determine the pharmacological profile of σ_1_R ligands, the most common method is to assess their potency to alleviate opioid analgesia. It has been well established that σ_1_R agonists reduce opioid analgesic activity, while σ_1_R antagonists have been demonstrated to enhance opioid analgesia in different pain models.

**Methods:**

In the present study, we evaluated the pharmacological profile of selected σ_1_R ligands using a novel object recognition (NOR) test, to see if any differences in cognitive functions between σ_1_R agonists and antagonists could be observed. We used the highly selective PRE-084 and S1RA as reference σ_1_R agonist and antagonist, respectively. Furthermore, compound KSK100 selected from our ligand library was also included in this study. KSK100 was previously characterized as a dual-targeting histamine H_3_/σ_1_R antagonist with antinociceptive and antiallodynic activity in vivo. Donepezil (acetylcholinesterase inhibitor and σ_1_R agonist) was used as a positive control drug.

**Results:**

Both tested σ_1_R agonists (donepezil and PRE-084) improved learning in the NOR test, which was not observed with the σ_1_R antagonists S1RA and KSK100.

**Conclusions:**

The nonlinear dose–response effect of PRE-084 in this assay does not justify its use for routine assessment of the functional activity of σ_1_R ligands.

**Graphical Abstract:**

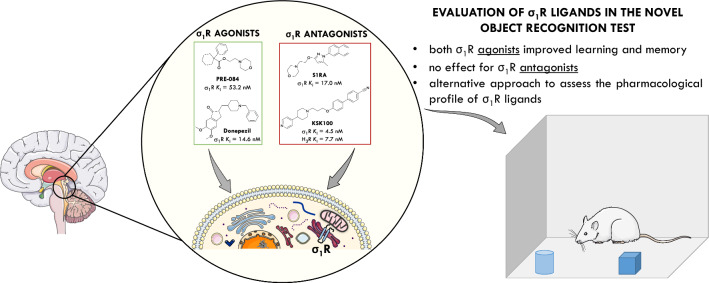

**Supplementary Information:**

The online version contains supplementary material available at 10.1007/s43440-023-00516-x.

## Introduction

In describing sigma (σ) receptors, the challenge is not that so little is known about these enigmatic proteins [1], but rather that much of what we thought we knew has turned out to be wrong. Sigma receptors, initially described as a subtype of opioid receptors, are now considered unique receptors. Pharmacological studies have distinguished two types of σ receptors, termed σ_1_ and σ_2_ [2, 3]. The σ_1_R is an unusual and poorly understood target, although it is thought to act as an intracellular chaperone [4] and is implicated in learning and memory, depression, anxiety, and schizophrenia [2, 5]. Sigma-1 antagonists are also effective in neuropathic pain [5] and, whilst the mechanism of action is not well understood, several studies suggest σ_1_ receptors regulate ion channel function (including NMDA/GluN receptors and K^+^/Ca^2+^ channels) [2]. Although the terms “agonist” and “antagonist” have been used to classify σ_1_ receptor ligands, an unambiguous definition of the functional activity (agonist versus antagonist) is often hard, especially since σ_1_R ligands are often not selective. Unfortunately, a lack of canonical functional assays for sigma receptors makes classifying the pharmacology of sigma-targeting ligands challenging.

**Fig. 1:**
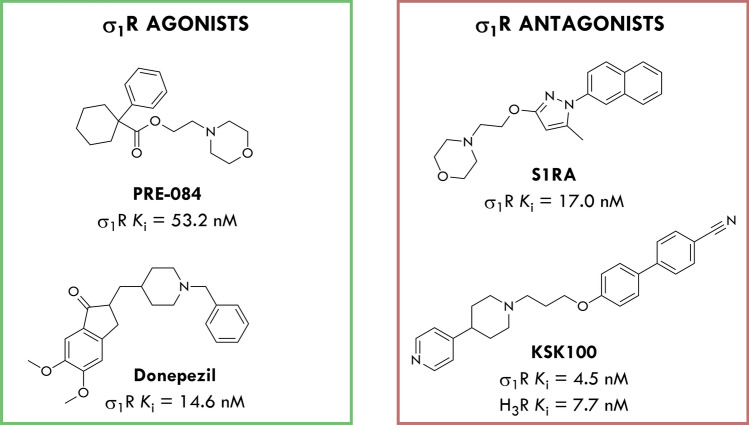
Structure and biological activity of compounds used in this work [3, 12, 13]

To date, some predictive approaches have been used to identify an agonist/antagonist profile of σ_1_R ligands, and the most common relate to their pain-related behavioral pharmacological evaluation. It has been well established that σ_1_R agonists (e.g.: pentazocine) reduce opioid analgesic activity, while σ_1_R antagonists (e.g.: S1RA, Fig. 1) have been demonstrated to enhance opioid analgesia in different pain models [6–8]. Ligands that induce the same phenotype as pentazocine are commonly accepted as σ_1_R agonists, while compounds that exhibit the same effects as S1RA are considered as antagonists. Importantly, σ_1_R antagonists also exhibit antinociceptive activity independently of opioids, e.g. in capsaicin and formalin models [2, 3]. In order to unambiguously determine the functional profile of σ_1_R ligands, in vitro studies were also performed, including a well-established phenytoin-based functional assay [9]. Previous studies have shown that phenytoin, a low-potent allosteric modulator for the σ_1_R, differentially modulates the affinity of σ_1_R ligands depending on their agonist *versus* antagonist functionality. Phenytoin potentiates the receptor binding affinity of σ_1_R agonists, however, it produces no effects or slightly reduced receptor binding affinity for σ_1_R antagonists [9]. In fact, the results of phenytoin-based functional assay correlate with the in vivo activity of tested compounds, but the weak point is that the differences in binding affinities with the phenytoin are very small compared to the values obtained for compounds without an allosteric modulator [9].

On the other hand, several studies have demonstrated the efficacy of σ_1_R agonists in some neuropsychiatric and neurodegenerative disorders [10]. For example, PRE-084 is a high affinity, selective σ_1_ agonist (Fig. 1) that produces an anti-amnesic effect in vivo [11]. Furthermore, donepezil (Fig. 1) has been reported to not only inhibit the acetylcholinesterase (AChE) but also activate σ_1_ receptors, which is postulated to contribute to its overall neuroprotective and anti-amnesic effects [12].

Noteworthy, some of the pharmacological effects of old-generation σ_1_R ligands were mediated by a naloxone-sensitive opioid receptor site, some by the phencyclidine (PCP) site within the NMDA receptor, and some by other, less well-defined mechanisms [1]. Thus, one of the key requirements for progress in this field is the development of new compounds having improved affinity and selectivity for the haloperidol-sensitive σ_1_R binding site.

Considering the above, in the present study we decided to evaluate the pharmacological profile of selected σ_1_R ligands using a novel object recognition (NOR) test, to see if any differences in cognitive functions between σ_1_R agonists and antagonists could be observed. We used the highly selective PRE-084 and S1RA as reference σ_1_R agonist and antagonist, respectively. Furthermore, compound KSK100 selected from our ligand library (Fig. 1) was also included in this study. KSK100 was previously characterized as a dual-targeting histamine H_3_/σ_1_R antagonist with antinociceptive and antiallodynic activity in vivo [13]. Donepezil (AchE inhibitor and σ_1_R agonist) was used as a positive control drug in the NOR test.

## Materials and methods

This study follows EQIPD (Enhancing Quality In Preclinical Data) guidelines [14]; certificate no. PL-INS-DBNDD-11-2021-1.

### Novel object recognition test

**Fig. 2:**
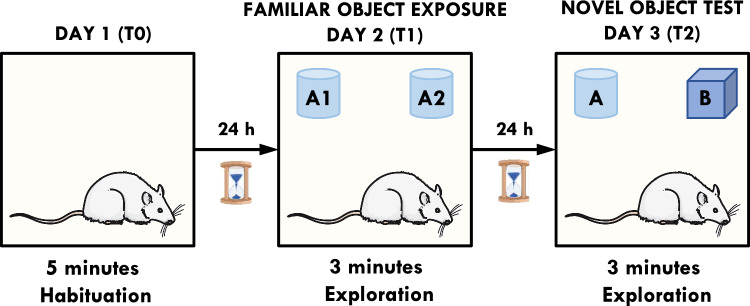
Experimental setup for the NOR task. The test takes place over 3 days. The first day is habituation (T0), in which a rat can explore the open field for 5 min. Day 2 is training (T1), in which the rat is allowed to explore the arena with 2 identical objects placed along the diagonal. Testing (T2) takes place 24 h after T1. Rats can explore the arena with one of the familiar objects and one novel object, placed along the diagonal

The novel object recognition test is now among the most commonly used cognitive behavioral tests in rodents. A rat is presented with two similar objects during the first session, and then one of the two objects is replaced by a new object during a second session (Fig. 2). The amount of time taken to explore the new object provides an index of recognition memory [15].

Rats were transferred to the experimental room for acclimation about 1 h before the test began. Animals were placed in an open field arena made of a dull gray plastic (66 × 56 × 30 cm) and tested in a dimly lit (25 lx). After each measurement, rats were return to a home cage and apparatus was cleaned and dried.

The procedure consisted of habituation to the arena (without any objects) for 5 min, 24 h before the test, and a test session comprised of two 3-min trials separated by an inter-trial interval of 24 h (ITI). During the first trial (familiarization, T1) two identical objects (A1 and A2) were presented in opposite corners, approximately 10 cm from the walls of the open field. In the second trial (recognition, T2) one of the objects was replaced by a novel one (A = familiar and B = novel). The objects used were the plastic bottles filled with sand and the glass beakers filled with gravel and. The objects were heavy enough not to be displaced by the animals and had a comparable height (~ 12 cm). The sequence of presentations and the location of the objects were randomly assigned to each rat and counterbalanced for each experimental group. The exploration was defined as a looking, licking, sniffing, or touching the object while sniffing, but not leaning against, standing, or sitting on the object. Any rat spending less than 5 s exploring the two objects within a 3 min trial was eliminated from the study. The exploration time and the distance traveled were measured using the Any-maze® video tracking system. Based on the exploration time (E) of two objects during T2, the discrimination index (DI) was calculated according to the formula: DI = (EB – EA)/(EA + EB).

### Animals

90 Male Sprague–Dawley rats (Charles River, Germany) weighing ~ 250 g at the arrival were housed in the standard laboratory cages, under standard colony A/C controlled conditions: room temperature 21 ± 2 °C, humidity (40–50%), 12-h light/dark cycle (lights on: 06:00) with ad libitum access to food and water. Rats were acclimatized for at least 7 days before the start of the experimental procedure. During this week animals were handled at least 5 times. Behavioral testing was carried out during the light phase of the light/dark cycle. Rats were randomly allocated to 9 experimental groups (each n = 10). Three rats from group KSK100 dose 1 and 3 mg/kg and PRE-084 0.3 mg/kg were excluded from the analysis due to exploration time < 5 s in trial T1. Once the experiment was completed rats were killed. All procedures and animals' maintenance were approved by the II Local Ethics Committee for Animal Experiments at the Maj Institute of Pharmacology, Polish Academy of Science, Kraków, Poland (ethical allowance 254/2022, 22-09-2022). All procedures followed the European Guidelines for animal welfare (2010/63/EU).

### Drugs

Donepezil hydrocholoride (Sigma Aldrich, US) was administered at the dose of 1 mg/kg, S1RA hydrochloride (Biosynth, UK) was administered at the doses of 15 and 30 mg/kg, PRE-084 hydrochloride (Angene, UK) was administered at the doses of 0.3 and 1 mg/kg. KSK100 was synthesized at the Department of Technology and Biotechnology of Drugs, Jagiellonian University Medical College (identity and purity were assessed by NMR and HPLC techniques, the purity was above 95%) and administered at the dose of 1, 3, and 10 mg/kg. All drugs were dissolved in the mixture of DMSO (5%) and Tween 80 (1%) in the 0.9% NaCl, which also served as a vehicle administered to controls. The doses of tested compounds were chosen based on our previous experiments on analgesic efficacy of sigma-1 receptor ligands [13]. Unfortunately, studies addressing the effects of S1RA and PRE-084 on memory in rats are limited. The selection of S1RA doses of 15 and 30 mg/kg is supported by the report of Ruiz-Leyva et al. [16]. The authors found that S1RA at neither the dose of 4, 16 nor 64 mg/kg affected Wistar rats’ memory in NOR, although at the doses of 16 and 64 mg/kg it demonstrated a central activity manifesting in reduced binge alcohol drinking. In mice, PRE-084 alleviated ischemic memory impairments at the doses of 1 and 3 mg/kg (examined in NOR, Morris water-maze and Y maze [17]) and reduced MK-801-induced memory impairments at the doses 0.3 and 1 mg/kg (examined in Y-maze and passive avoidance test [18, 19]), but not 0.1 or 3 mg/kg [20]. Maurice reported that PRE-084 at the dose of 0.5 mg/kg reduced age-related memory deficits in Morris water-maze test in Wistar rats [21]. The positive control group was selected based on our earlier preliminary studies and literature reports [22, 23] that found donepezil 1 mg/kg effective in NOR (ITI = 24h) in Sprague Dawley rats. All drugs were injected to rats *ip* 30 min before trial T1.

### Statistical analysis

We used G*Power v. 3.1.9.6 for a priori effect and sample size analyses; a sufficient number of animals was estimated at least 7 per group (*p* = 0.05, Cohen’s f = 0.8129). Due to the exclusion criterion (exploration < 5 s) 10 animals per group was assumed. For statistical evaluation, we employed one-way ANOVA followed by Dunnett’s post hoc test. All datasets passed the assumption of parametric analysis, here examined by the test of Shapiro–Wilk test of normal data distribution and Levene’s test for variances’ equality. We used IBM SPSS Statistics v. 25 for statistical examination.

## Results

**Fig. 3:**
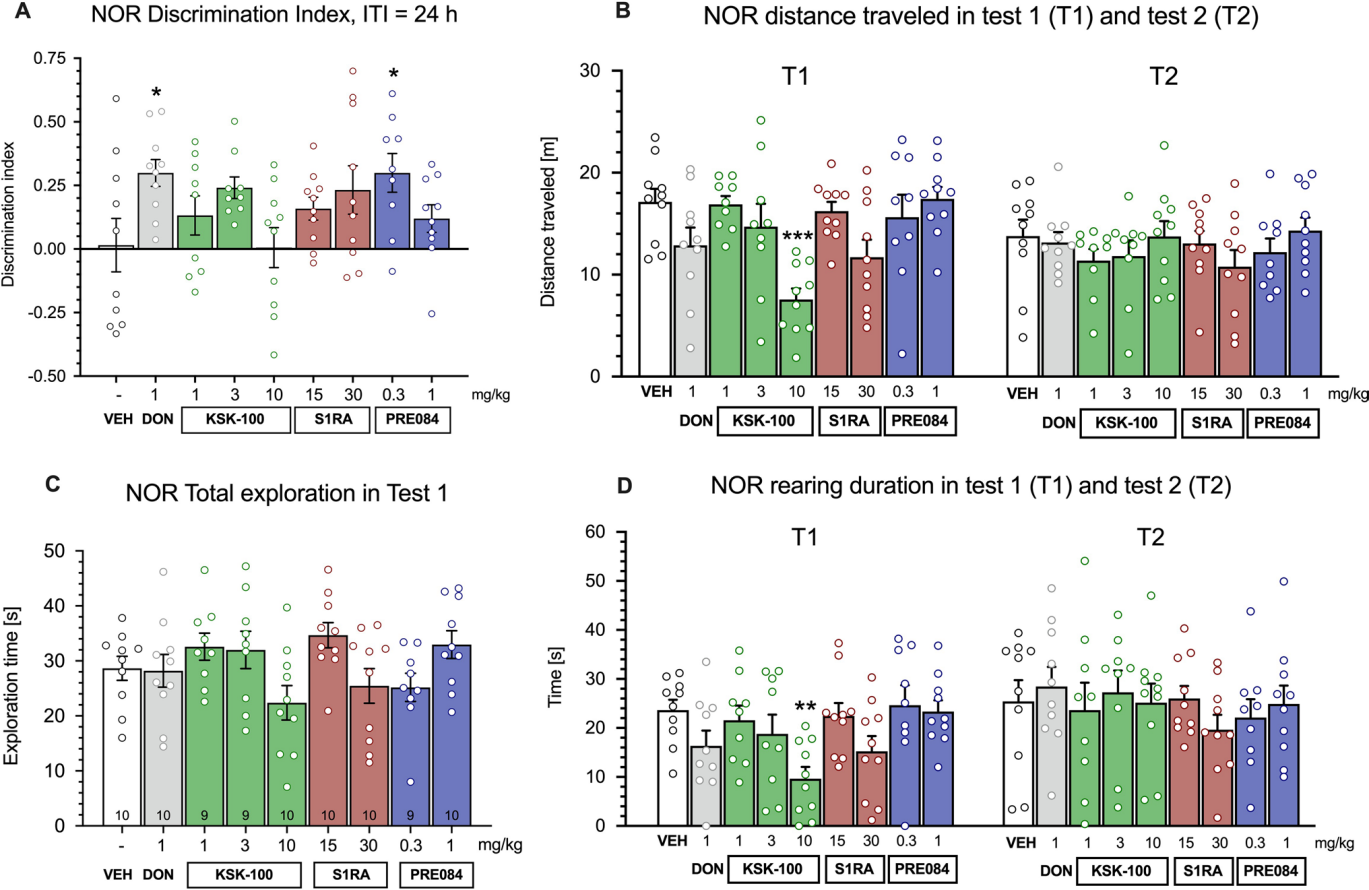
Effects of KSK100, donepezil, SR1A and PRE084 on object recognition (**A**), distance traveled (**B**), exploration time in familiarization (T1) phase (**C**) and rearing time (**D**) in the novel object recognition (NOR) test in rats. Drugs were administered *ip* 30 min before NOR familiarization trial. Data are expressed as mean ± SEM. Statistical analysis: Shapiro–Wilk test for normality, Levene’s test for homogeneity of variance and 1-way ANOVA followed by Dunnett’s post hoc test for between-subject differences, *p* < 0.05 was considered statistically significant. Symbols: (* *p* < 0.05, ** *p* < 0.01, *** *p* < 0.001) indicate a statistical significance as compared to vehicle-treated rats. The number of animals per group was 9 or 10 and is shown at the bottom of Fig. 3C. *DON* donepezil, *ITI* inter-trial interval, *NOR* novel object recognition test, *VEH* vehicle

**Fig. 4:**
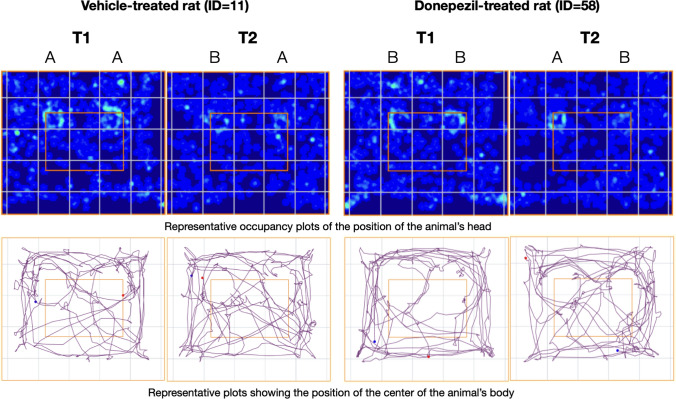
Representative plots of animal head position occupancy (top panel) and animal movement trajectory (bottom panel). Plots were generated using AnyMaze software. T1 and T2 represent familiarization and recognition phase, respectively. Letters A and B represent types of the objects presented in T1 and T2. The objects were placed in the left and right upper corners of the inner square marked at the snapshots. Plots for all subjects are available in the supplementary materials

One-way ANOVA demonstrated that the discrimination index differed across tested groups (F_8,78_ = 2.292, *p* = 0.029). Dunnett’s post hoc found that animals treated with donepezil 1 mg/kg and PRE-084 0.3 mg/kg presented a significantly increased discrimination index than controls (*p* < 0.05) (Fig. 3). Total exploration time during T1 was at a similar level for all tested groups (F_8,78_ = 1.984; *p* = ns). However, KSK100 at the highest tested dose of 10 mg/kg reduced the rearing time (F_8,78_ = 2.864, *p* = 0.008) and distance traveled (F_8,78_ = 4.596, *p* < 0.0001) in T1 (i.e. 30 min after *ip* injection) as compared to control animals (*p* < 0.01). No significant differences in locomotor activity (rearing and distance) were found 24 h after injection in T2 (Fig. 3). Representative plots of animal head position occupancy and movement trajectories are shown in Fig. 4.

## Discussion

Both tested σ_1_R agonists (donepezil and PRE-084) have been shown to improve learning and memory in the NOR test, which was not observed with the σ_1_R antagonists S1RA and KSK100. However, it should be emphasized that donepezil has a more complex mechanism of action than PRE-084 and was only used as a positive control in the NOR test. Nevertheless, compared to vehicle, administration of σ_1_R agonists significantly enhanced memory, however, the effect of PRE-084 was observed only at a lower dose. This compound was active in the NOR test at 0.3 mg/kg but completely lost activity at 1 mg/kg. Interestingly, PRE-084 has been used in animal models of several central nervous system (CNS) pathologies, particularly related to cognitive functions such as learning and memory, and administered in mice in a wide range of doses (from 0.1 to 64.0 mg/kg) [24]. In fact, a biphasic nonlinear dose–response relation has been previously reported for cognitive enhancers such as AChE inhibitors [25, 26], nootropics [27] and α7 nicotinic acetylcholine receptor modulators [28]. This hormesis has been previously noted also for PRE-084 in mice [18, 20] and most likely underlies our finding of the potential procognitive effect of this ligand at the dose of 0.3 mg/kg but not 1 mg/kg in rats. Moreover, it has been proposed that σ_1_R agonists fail to improve cognition at higher doses due to a non-selective σ_2_R activation or NMDA receptor modulation [29]. However, with the high selectivity of PRE-084, we do not suspect an interaction with another biological target that could attenuate its pharmacological effect at a higher dose.

Since our study follows the EQIPD guidelines (a system that aims to optimize the robustness and reliability of preclinical biomedical research) and was carefully designed in this particular way, it is not possible to test a wider range of PRE-084 doses and compare the results of two separate experiments. Moreover, the doses, routes, and time of drug administration were selected based on our previous study [13] and literature data [16, 17, 19, 30]. In fact, they indicate doses of 0.3 and 1 mg/kg for PRE-084 as potentially effective in such an experiment [17, 19, 20].

In the present study, neither KSK100, donepezil, SR1A nor PRE-084 affected exploration time in NOR familiarization (T1) phase. However, one of the tested σ_1_R antagonists KSK100 at the dose of 10 mg/kg significantly affected the locomotor activity (rearing and distance) in T1. The high sedative activity is considered an undesirable property, which may lead to an incorrect or ambiguous interpretation of the in vivo results, and may limit the potential clinical application of new drug candidates. Interestingly, in our previous study, KSK100 significantly decreased spontaneous locomotor activity, but at a much higher dose of 30 mg/kg and in a different animal model [13]. At doses of 5 and 15 mg/kg, we observed no significant effect on locomotor activity. In addition, KSK100 appears to be a potent H_3_R antagonist, which could be important due to the involvement of histaminergic system(s) in the pathophysiology of psychiatric disorders [31]. For instance, the learning and memory-facilitating potential of plentiful H_3_R antagonists have been shown. However, in our study, KSK100 did not significantly influence object recognition.

In conclusion, despite differences in the effects on learning and memory between σ_1_R agonists and antagonists, the nonlinear dose–response effect of the reference compound PRE-084 does not justify the use of the NOR test for routine assessment of functional activity of σ_1_R ligands. It appears that the tests involving opioid-induced analgesia allow for a more precise assessment of their pharmacological profile and determination of whether they are σ_1_R agonists or antagonists.

### Supplementary Information

Below is the link to the electronic supplementary material.

Supplementary file1 (PDF 119 KB)

Supplementary file2 (PDF 144 KB)

Supplementary file3 (PDF 100 KB)

Supplementary file4 (PDF 143 KB)

Supplementary file5 (PDF 102 KB)

Supplementary file6 (PDF 143 KB)

Supplementary file7 (PDF 86 KB)

Supplementary file8 (PDF 143 KB)

Supplementary file9 (PDF 114 KB)

Supplementary file10 (PDF 147 KB)

Supplementary file11 (PDF 97 KB)

Supplementary file12 (PDF 147 KB)

Supplementary file13 (PDF 83 KB)

Supplementary file14 (PDF 140 KB)

Supplementary file15 (PDF 72 KB)

Supplementary file16 (PDF 138 KB)

Supplementary file17 (PDF 81 KB)

Supplementary file18 (PDF 142 KB)

Supplementary file19 (PDF 71 KB)

Supplementary file20 (PDF 142 KB)

Supplementary file21 (PDF 94 KB)

Supplementary file22 (PDF 144 KB)

Supplementary file23 (PDF 82 KB)

Supplementary file24 (PDF 143 KB)

Supplementary file25 (XLSX 13 KB)

## Data Availability

All data generated or analyzed during this study are included in this published article and its supplementary information files.

## Abbreviations

AChE: Acetylcholinesterase
CNS: Central nervous system
DI: Discrimination index
DMSO: Dimethyl sulfoxide
DON: Donepezil
E: Exploration time
H_3_R: Histamine H_3_ receptor
ITI: Inter-trial interval
NMDA: N-methyl-D-aspartate receptor
NOR test: Novel object recognition test
σ_1_R: Sigma-1 receptor
SEM: Standard error of the mean
PCP: Phencyclidine
σ_2_R: Sigma-2 receptor
VEH: Vehicle

## References

[1] Chavkin C (1990). The sigma enigma: biochemical and functional correlates emerge for the haloperidol-sensitive sigma binding site. Trends Pharmacol Sci.

[2] Cobos EJ, Entrena JM, Nieto FR, Cendán CM, Del Pozo E (2008). Pharmacology and therapeutic potential of sigma(1) receptor ligands. Curr Neuropharmacol.

[3] Szczepańska K, Kuder KJ, Kieć-Kononowicz K (2021). Dual-targeting Approach on Histamine H_3_ and Sigma-1 Receptor Ligands as Promising Pharmacological Tools in the Treatment of CNS-linked Disorders. Curr Med Chem.

[4] Hayashi T, Tsai SY, Mori T, Fujimoto M, Su TP (2011). Targeting ligand-operated chaperone sigma-1 receptors in the treatment of neuropsychiatric disorders. Expert Opin Ther Targets.

[5] Zamanillo D, Romero L, Merlos M, Vela JM (2013). Sigma 1 receptor: a new therapeutic target for pain. Eur J Pharmacol.

[6] Chien CC, Pasternak GW (1995). Sigma antagonists potentiate opioid analgesia in rats. Neurosci Lett.

[7] Vidal-Torres A, de la Puente B, Rocasalbas M, Touriño C, Bura SA, Fernández-Pastor B (2013). Sigma-1 receptor antagonism as opioid adjuvant strategy: enhancement of opioid antinociception without increasing adverse effects. Eur J Pharmacol.

[8] Szczepańska K, Podlewska S, Dichiara M, Gentile D, Patamia V, Rosier N (2022). Structural and Molecular Insight into Piperazine and Piperidine Derivatives as Histamine H_3_ and Sigma-1 Receptor Antagonists with Promising Antinociceptive Properties. ACS Chem Neurosci.

[9] Dichiara M, Ambrosio FA, Barbaraci C, González-Cano R, Costa G, Parenti C (2023). Synthesis, Computational Insights, and Evaluation of Novel Sigma Receptors Ligands. ACS Chem Neurosci.

[10] Hayashi T (2015). Sigma-1 receptor: the novel intracellular target of neuropsychotherapeutic drugs. J Pharmacol Sci.

[11] Maurice T, Phan VL, Noda Y, Yamada K, Privat A, Nabeshima T (1999). The attenuation of learning impairments induced after exposure to CO or trimethyltin in mice by sigma (sigma) receptor ligands involves both sigma1 and sigma2 sites. Br J Pharmacol.

[12] Meunier J, Ieni J, Maurice T (2006). The anti-amnesic and neuroprotective effects of donepezil against amyloid beta25-35 peptide-induced toxicity in mice involve an interaction with the sigma1 receptor. Br J Pharmacol.

[13] Szczepańska K, Karcz T, Dichiara M, Mogilski S, Kalinowska-Tłuścik J, Pilarski B (2023). Dual Piperidine-Based Histamine H _3_ and Sigma-1 Receptor Ligands in the Treatment of Nociceptive and Neuropathic Pain. J Med Chem.

[14] Bespalov A, Bernard R, Gilis A, Gerlach B, Guillén J, Castagné V (2021). Introduction to the EQIPD quality system. Elife.

[15] Leger M, Quiedeville A, Bouet V, Haelewyn B, Boulouard M, Schumann-Bard P, Freret T (2013). Object recognition test in mice. Nat Protoc.

[16] Ruiz-Leyva L, Salguero A, Morón I, Portillo-Salido E, Cendán CM, Pautassi RM (2020). Sigma-1 antagonism inhibits binge ethanol drinking at adolescence. Drug Alcohol Depend.

[17] Xu Q, Ji XF, Chi TY, Liu P, Jin G, Gu SL (2015). Sigma 1 receptor activation regulates brain-derived neurotrophic factor through NR2A-CaMKIV-TORC1 pathway to rescue the impairment of learning and memory induced by brain ischaemia/reperfusion. Psychopharmacology.

[18] Maurice T, Su TP, Parish DW, Nabeshima T, Privat A (1994). PRE-084, a sigma selective PCP derivative, attenuates MK-801-induced impairment of learning in mice. Pharmacol Biochem Behav.

[19] Martin P, de Witte PAM, Maurice T, Gammaitoni A, Farfel G, Galer B (2020). Fenfluramine acts as a positive modulator of sigma-1 receptors. Epilepsy Behav.

[20] Martin P, Maurice T, Gammaitoni A, Farfel G, Boyd B, Galer B (2022). Fenfluramine modulates the anti-amnesic effects induced by sigma-1 receptor agonists and neuro(active)steroids in vivo. Epilepsy Behav.

[21] Maurice T (2001). Beneficial Effect of the s Receptor Agonist PRE-084 against the Spatial 1 Learning Deficits in Aged Rats. Eur J Pharmacol.

[22] Giorgetti M, Gibbons JA, Bernales S, Alfaro IE, Drieu La Rochelle C, Cremers T (2010). Cognition-enhancing properties of Dimebon in a rat novel object recognition task are unlikely to be associated with acetylcholinesterase inhibition or N-methyl-D-aspartate receptor antagonism. J Pharmacol Exp Ther.

[23] Mørk A, Montezinho LP, Miller S, Trippodi-Murphy C, Plath N, Li Y, Gulinello M, Sanchez C (2013). Vortioxetine (Lu AA21004), a novel multimodal antidepressant, enhances memory in rats. Pharmacol Biochem Behav.

[24] Motawe ZY, Abdelmaboud SS, Cuevas J, Breslin JW (2020). PRE-084 as a tool to uncover potential therapeutic applications for selective sigma-1 receptor activation. Int J Biochem Cell Biol.

[25] Braida D, Paladini E, Griffini P, Lamperti M, Maggi A, Sala M (1996). An inverted U-shaped curve for heptylphysostigmine on radial maze performance in rats: comparison with other cholinesterase inhibitors. Eur J Pharmacol.

[26] Van Dam D, Abramowski D, Staufenbiel M, De Deyn PP (2005). Symptomatic effect of donepezil, rivastigmine, galantamine and memantine on cognitive deficits in the APP23 model. Psychopharmacology.

[27] Schindler U, Rush DK, Fielding S (1984). Nootropic Drugs: Animal Models for Studying Effects on Cognition. Drug Dev Res.

[28] Wang X, Daley C, Gakhar V, Lange HS, Vardigan JD, Pearson M (2020). Pharmacological Characterization of the Novel and Selective *α*7 Nicotinic Acetylcholine Receptor-Positive Allosteric Modulator BNC375. J Pharmacol Exp Ther.

[29] Maurice T (2021). Bi-phasic dose response in the preclinical and clinical developments of sigma-1 receptor ligands for the treatment of neurodegenerative disorders. Expert Opin Drug Discov.

[30] Alachkar A, Łażewska D, Kieć-Kononowicz K, Sadek B (2017). The Histamine H3 Receptor Antagonist E159 Reverses Memory Deficits Induced by Dizocilpine in Passive Avoidance and Novel Object Recognition Paradigm in Rats. Front Pharmacol.

[31] Passani MB, Lin JS, Hancock A, Crochet S, Blandina P (2004). The histamine H3 receptor as a novel therapeutic target for cognitive and sleep disorders. Trends Pharmacol Sci.

