# Supplementary material for: Novel object recognition test as an alternative approach to assessing the pharmacological profile of sigma-1 receptor ligands
Source: Pharmacol Rep. 2023 Aug 12;75(5):1291–8. doi: 10.1007/s43440-023-00516-x (PMC10539447; doi:10.1007/s43440-023-00516-x)
Supplement: Supplementary file 12 — Supplementary file12 (PDF 147 KB) [file 43440_2023_516_MOESM12_ESM.pdf]

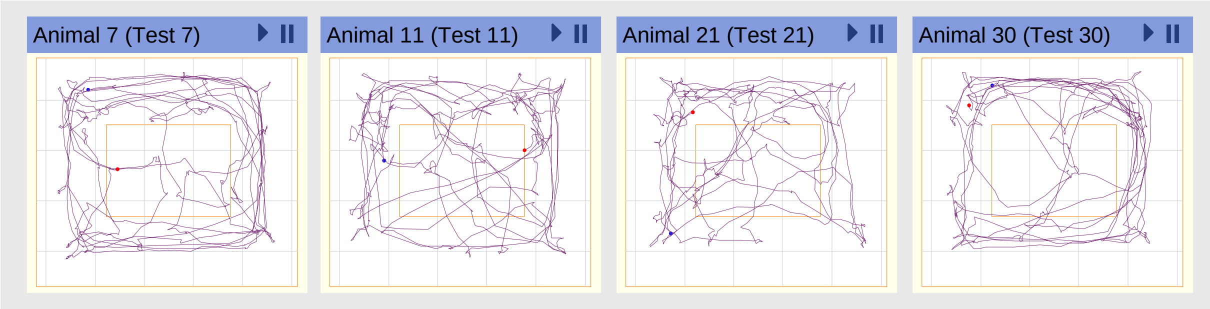

Figure 1. Track plots showing the position of the centre of the animal for tests where Treatment is VEHICLE.

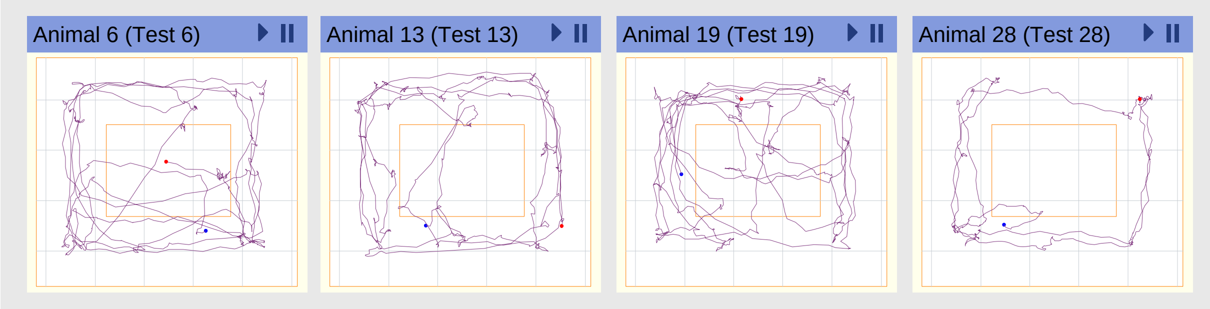

Figure 2. Track plots showing the position of the centre of the animal for tests where Treatment is KSK100_10.

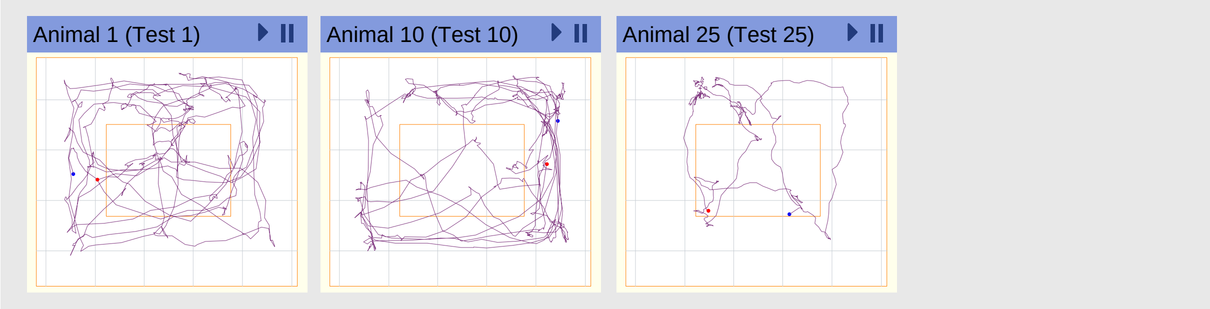

Figure 3. Track plots showing the position of the centre of the animal for tests where Treatment is KSK100_3.

©

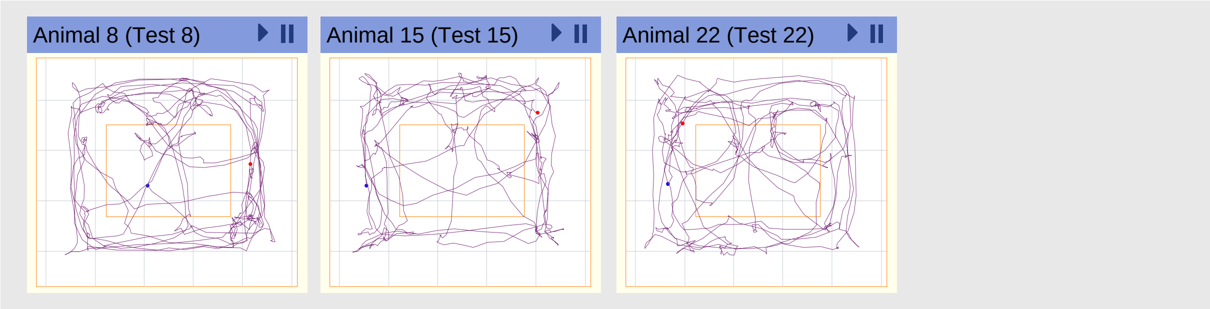

Figure 4. Track plots showing the position of the centre of the animal for tests where Treatment is KSK100_1.

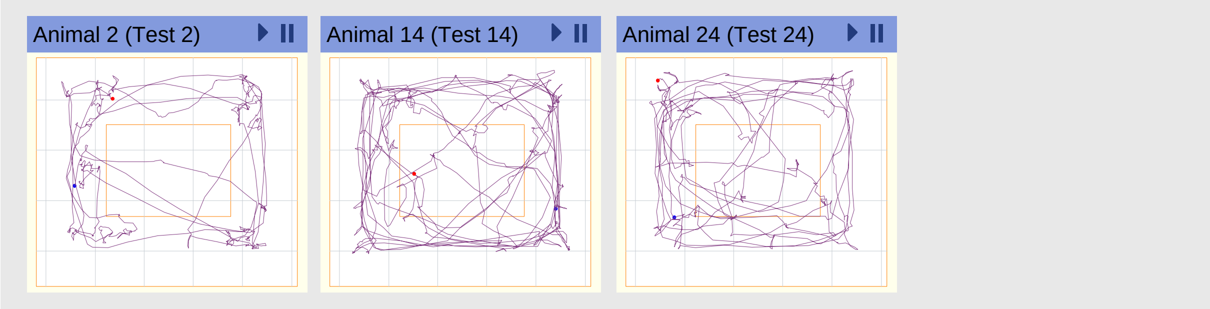

Figure 5. Track plots showing the position of the centre of the animal for tests where Treatment is PRE084_1.

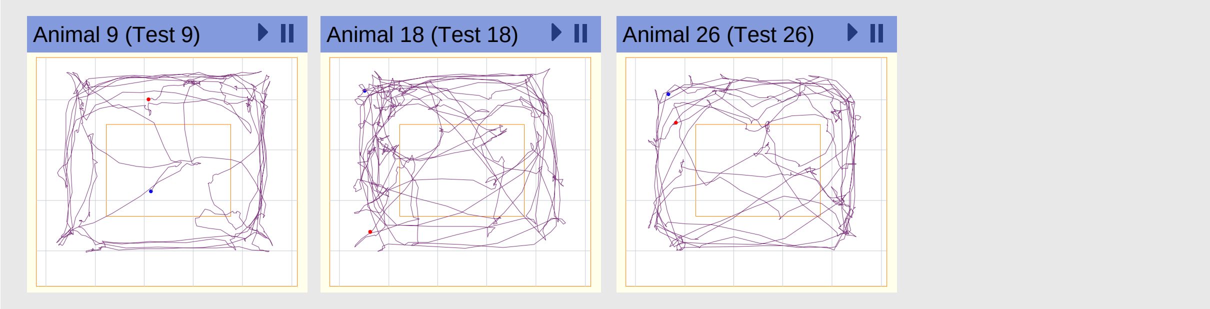

Figure 6. Track plots showing the position of the centre of the animal for tests where Treatment is PRE084_03.

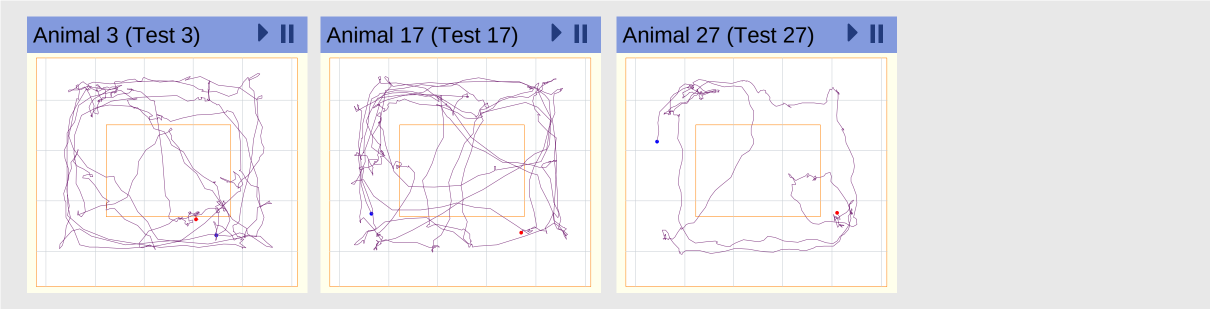

Figure 7. Track plots showing the position of the centre of the animal for tests where Treatment is SR1A_30.

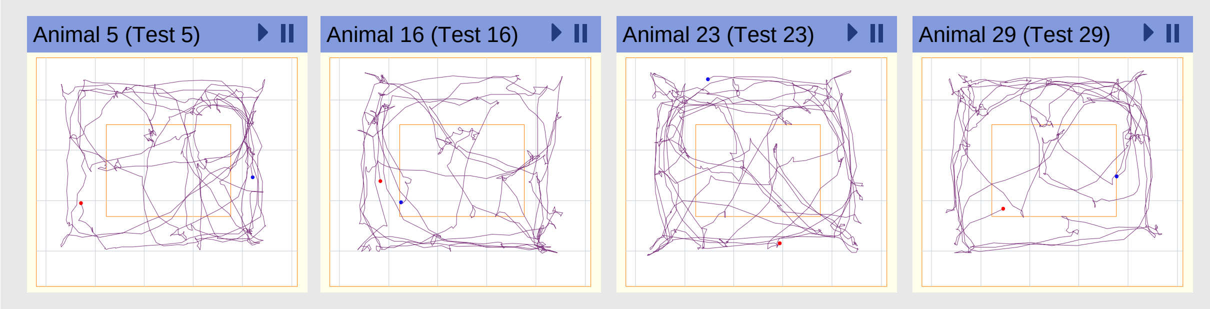

Figure 8. Track plots showing the position of the centre of the animal for tests where Treatment is SR1A_15.

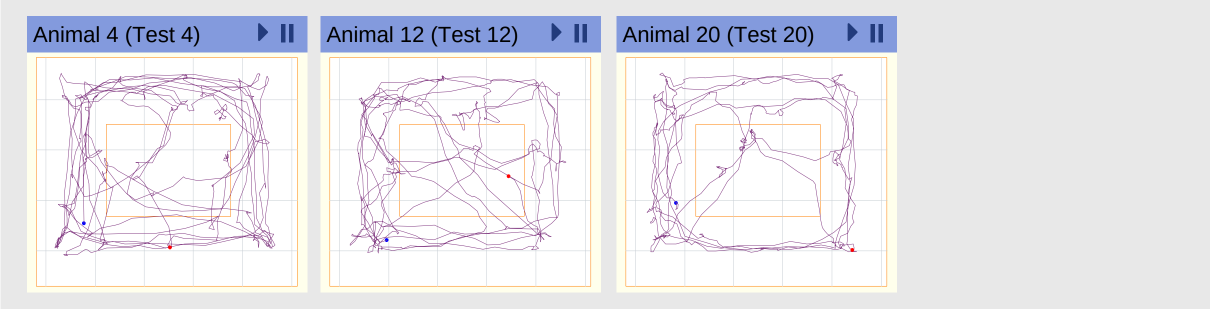

Figure 9. Track plots showing the position of the centre of the animal for tests where Treatment is DONEPEZIL_1.

Notes:

1. The following symbols have been used in these plots:

- *Track start*
- *Track end*
- *Pause on*
- *Pause off*
