# Supplementary material for: Novel object recognition test as an alternative approach to assessing the pharmacological profile of sigma-1 receptor ligands
Source: Pharmacol Rep. 2023 Aug 12;75(5):1291–8. doi: 10.1007/s43440-023-00516-x (PMC10539447; doi:10.1007/s43440-023-00516-x)
Supplement: Supplementary file 19 — Supplementary file19 (PDF 71 KB) [file 43440_2023_516_MOESM19_ESM.pdf]

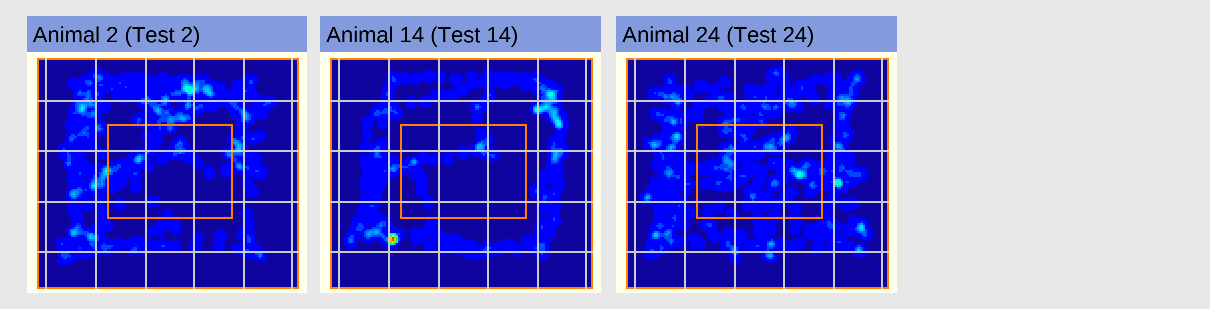

Figure 1. Occupancy plots of the position of the centre of the animal for tests where Treatment is VEHICLE.

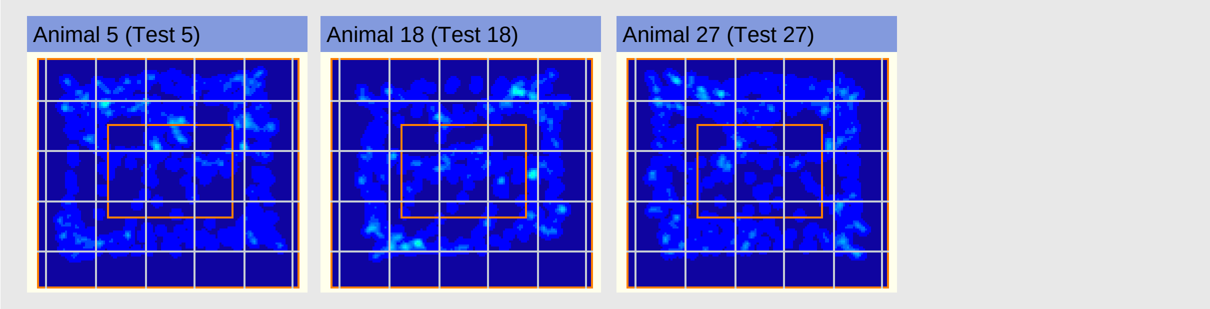

Figure 2. Occupancy plots of the position of the centre of the animal for tests where Treatment is KSK100_10.

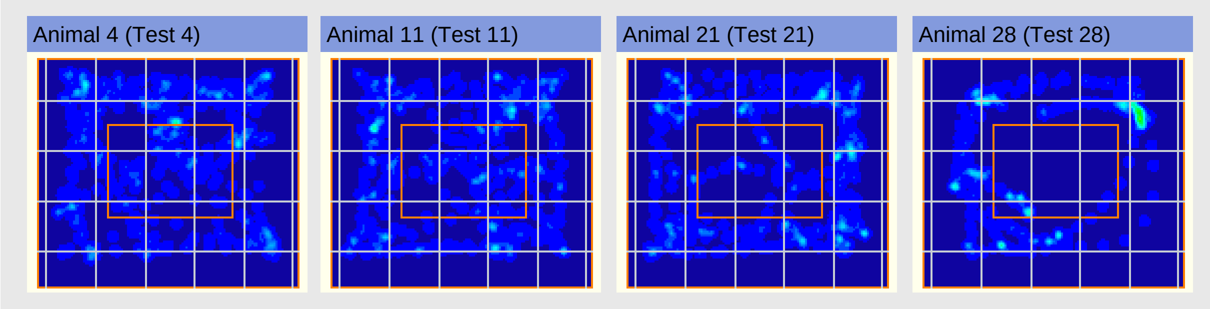

Figure 3. Occupancy plots of the position of the centre of the animal for tests where Treatment is KSK100_3.

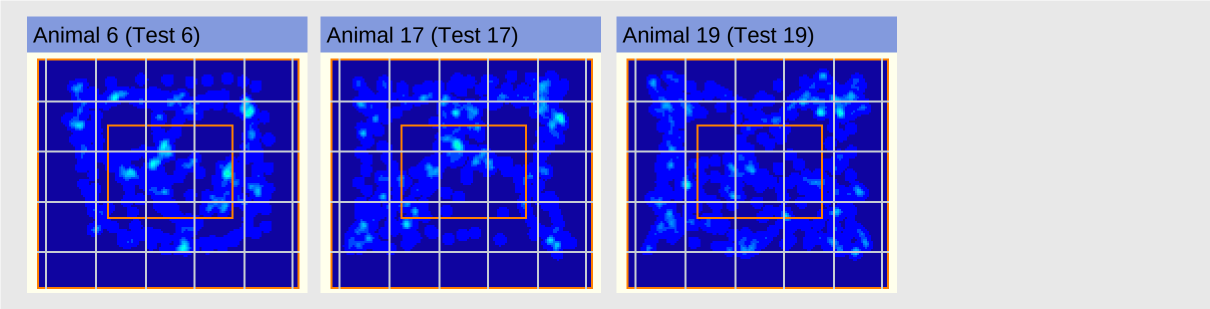

Figure 4. Occupancy plots of the position of the centre of the animal for tests where Treatment is KSK100_1.

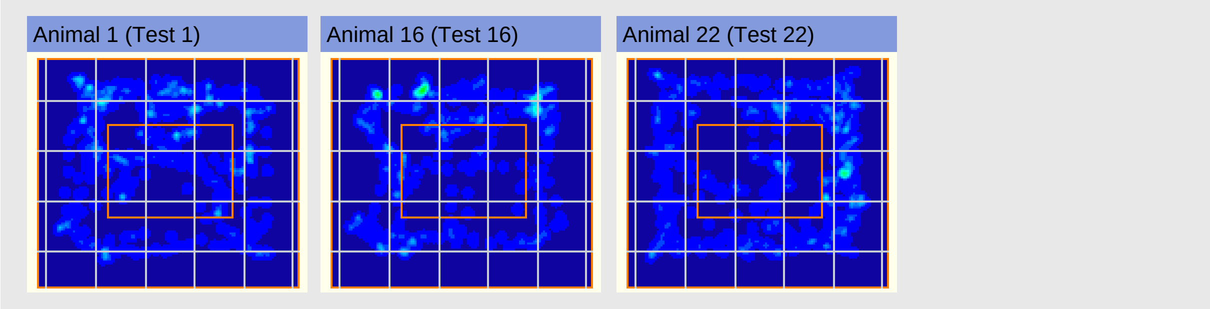

Figure 5. Occupancy plots of the position of the centre of the animal for tests where Treatment is PRE084_1.

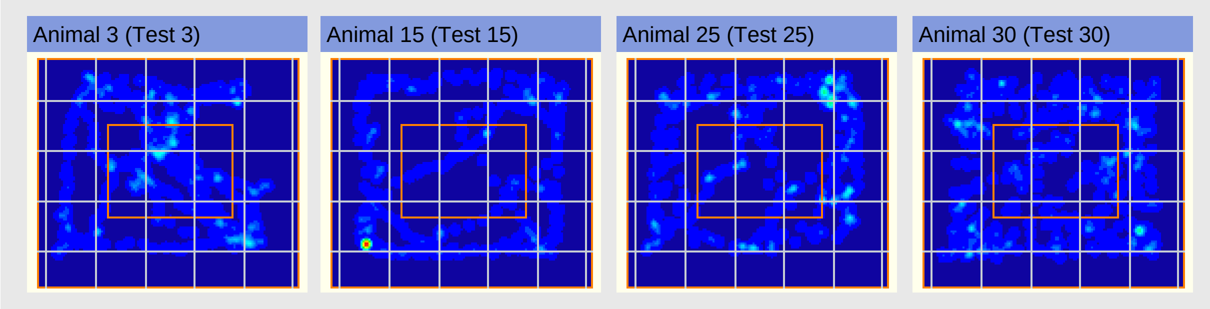

Figure 6. Occupancy plots of the position of the centre of the animal for tests where Treatment is PRE084_03.

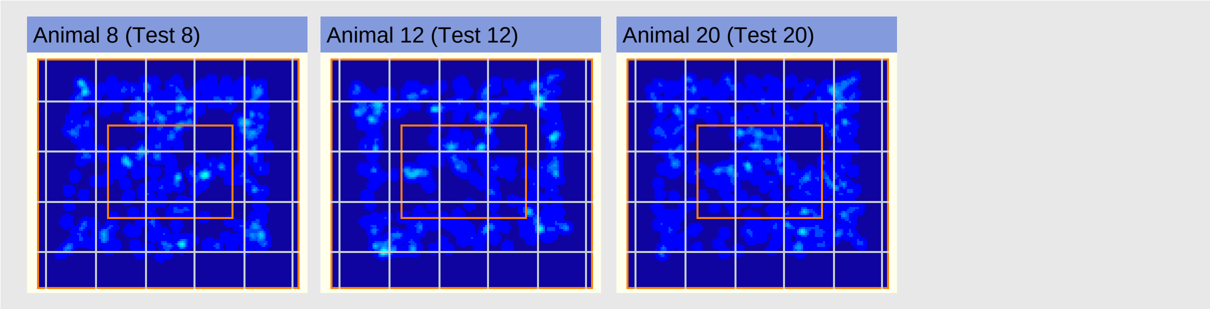

Figure 7. Occupancy plots of the position of the centre of the animal for tests where Treatment is SR1A_30.

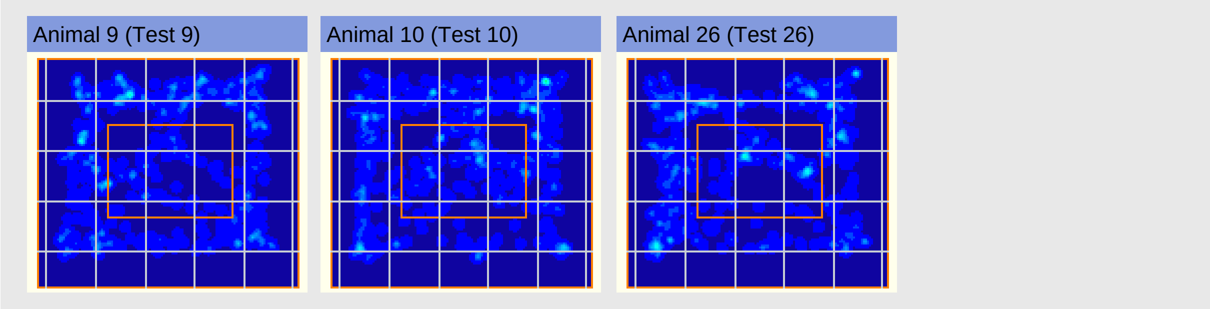

Figure 8. Occupancy plots of the position of the centre of the animal for tests where Treatment is SR1A_15.

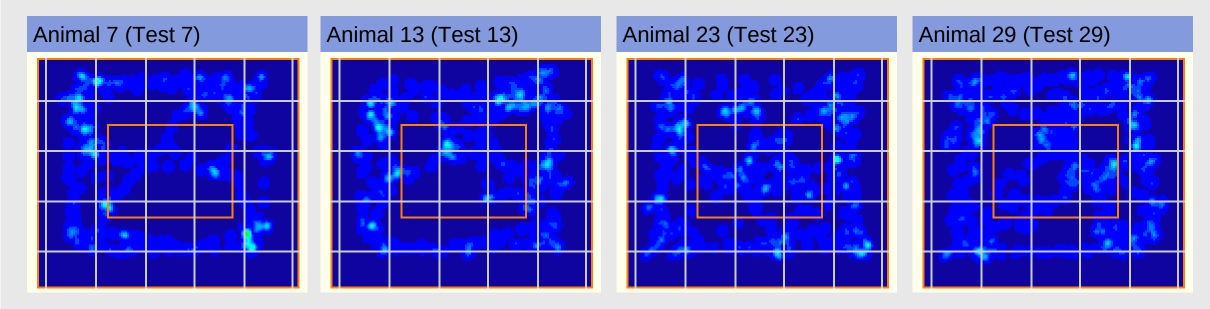

Figure 9. Occupancy plots of the position of the centre of the animal for tests where Treatment is DONEPEZIL_1.

Notes:

1. *The value for the maximum occupancy in the above plots is based on the maximum of the centre and, where relevant, head positions, across all the tests in the entire experiment. This makes it possible to compare the plots of different tests.*

©
